# Passive Immunity Transfer in Water Buffaloes (*Bubalus bubalis*)

**DOI:** 10.3389/fvets.2020.00247

**Published:** 2020-06-17

**Authors:** Damazio Campos de Souza, Daniela Gomes da Silva, Lana Cristine Coelho Fonseca, Letícia de Castro Fiori, Bruno Moura Monteiro, Otávio Bernardes, Rinaldo Batista Viana, José Jurandir Fagliari

**Affiliations:** ^1^Faculdade de Ciências Agrárias e Veterinárias, Universidade Estadual Paulista, Jaboticabal, Brazil; ^2^Institute of Health and Animal Production, Universidade Federal Rural da Amazônia, Paragominas, Brazil; ^3^Sítio Paineiras da Ingaí, Alambari, Brazil; ^4^Institute of Health and Animal Production, Universidade Federal Rural da Amazônia, Belém, Brazil

**Keywords:** calf, colostrum, murrah, IgG, neonate, newborn

## Abstract

This study aimed to evaluate passive immunity transfer in healthy buffalo calves. Colostrum samples from heifers (without previous calving) and primiparous and pluriparous dams and blood samples from their offspring were obtained at calving, before colostrum intake, and at 24, 48, and 72 h after calving for determination of serum activities of gammaglutamyltransferase and alkaline phosphatase and serum concentrations of total protein (TP), immunoglobulin A (IgA) and IgG, and lactoferrin. The results were analyzed as repeated measures, and differences were considered statistically significant at *P* ≤ 0.05. Considering that the buffalo calves were born hypogammaglobulinemic (4.23 ± 0.33 mg/ml) and, at 24 h, the mean serum concentration of IgG was 34.5 ± 1.48 mg/ml, passive immunity transfer was successful. Moreover, colostrum IgG concentrations at 0 h were correlated with serum IgG concentrations at 24 h in buffalo calves. Additionally, TP concentrations were highly correlated with IgG in both colostrum at calving and blood in calves at 24 h. TP is recommended as a reliable indirect parameter to evaluate both colostrum quality and passive immunity transfer in buffalo calves.

## Introduction

The water buffalo (*Bubalus bubalis*) is a species that is important to Asian, Mediterranean, and South American societies because it is essential to the livelihood of many families in developing countries as a source of income and food safety. The most recent reports estimate a world population of 199 million animals, with 193,795,922 heads in Asian countries (1). In South America, Brazil is the largest producer, with 1,381,345 heads, a milk production of 100 million L/year, and an estimated market value of USD 300 million (2).

As ruminants, buffaloes are dependent on immunoglobulin (Ig) present in the colostrum because they are born agammaglobulinemic or hypogammaglobulinemic (3). IgG is responsible for calf immunity during the first month of life and represents 86% of total Igs in buffalo colostrum, with the additional aid of IgA (8%) and IgM (6%) (4). The predominance of IgG is due to active and selective receptors in the epithelium of the mammary gland. These same receptors are present in the intestinal epithelial cells of calves and carry IgG through endocytosis in blood circulation (5).

The most efficient way to ensure low mortality levels in bovine calves is to verify that passive immunity transfer (PIT) occurs, more specifically, that calves have absorbed at least 20–25 mg/ml of colostral IgG. A failure of PIT (FPIT) prevalence of <10% is a reasonable goal in ruminants (6). The odds of FPIT are higher when there is no on-farm routine screening, and benchmarking PIT values has been shown to enhance production outcomes as better management practices were adopted on dairy farms (7, 8). The measurement of IgG concentrations, both in the colostrum and blood serum of calves, serves as a tool to evaluate colostrum management.

Despite the importance of the species in world dairy production, the available knowledge regarding PIT in buffaloes remains scarce. Therefore, the aim of this study was to evaluate PIT that occurs in newborn buffaloes by measuring serum concentrations of IgG in both colostrum and calf blood and to evaluate associations among the studied parameters.

## Materials and Methods

### Farm Conditions and Animals

Seventy-two healthy Murrah females (15 heifers without previous calving; 18 primiparous buffaloes; 11 multiparous buffaloes with two or three parities; 15 multiparous buffaloes with four or five parities; and 13 multiparous buffaloes with more than six parities), with an average weight after delivery of 627 ± 91 kg, raised in a farm of 116 ha with a semi-extensive system, with an average milk production of 2,650 ± 635 kg in 300 days/dam with an average of 6.40% of milk fat and 4.24% of milk protein, located in São Paulo state, Brazil (23° 34S, 47°49 W) were used in the study. All animals were clinically healthy and had free access to water and *Brachiaria* spp. pastures and were fed with a diet consisting of corn silage, soybeans, and cottonseed twice per day. They were kept under an average environmental temperature of 23 ± 7°C. All females had a dry period of 70 days and were vaccinated against brucellosis, clostridiosis, hemorrhagic septicemia, bovine viral diarrhea, leptospirosis, and foot and mouth disease. No vaccination was done during late gestation.

All their calves (33 females, 39 males; average weight of 38.0 ± 5.55 kg), born from natural breeding via eutocic, were also part of this study. All animals were born in a maternity pen under careful observation to prevent the calves from sucking off another mother. Immediately after deliverance and after the dams recognized their calves, they were separated, and the birth and weight of the calves were registered. After first feeding, the calves' umbilical cords were treated with 2% iodine solution. The calves were free to suckle on their dam during their first 5 days of life. Only calves that had suckled <4 h after calving entered the study. Animals underwent thorough daily physical examination and were considered clinically healthy when they did not present any alterations on physical examination (9). Sampling of colostrum and calf's blood occurred at calving, before they could suckle (0 h), and at 24, 48, and 72 h after calving. All animals were raised in the same conditions.

### Sampling and Chemistry Analysis

A 10-ml blood sample from calves was collected by jugular venipuncture after local antisepsis. A vacuum collection system in siliconized tubes without anticoagulant (Vacutainer, Becton Dickinson, Franklin Lakes, NJ, USA) was used. Blood samples were clotted at room temperature for 30 min, and serum was collected after centrifugation at 2,000 rpm (Excelsa Baby −208N, Fanem, São Paulo, Brazil) on field for 10 min, divided into 2.0-ml aliquots, stored in microtubes, and maintained at −20 °C until the laboratory tests were performed.

The colostrum samples were pooled from all mammary quarters and stored in 50-ml polypropylene tubes put and maintained at −20°C. For colostrum whey separation, renin solution was added (Coalho Estrella, Chr. Hansen Brazil Ind. and Com. LTDA, Valinhos, Brazil) in an amount corresponding to 5% of the volume of milk secretion. The samples were placed in a 37°C water bath for 20 min until formation and retraction of the clot. Subsequently, samples were centrifuged at 5,000 rpm for 20 min in a refrigerated (4°C) centrifuge (Centrifuge 5804 R, Eppendorf, Hamburg, Germany) at the laboratory. After centrifugation, the colostrum whey was aspirated and stored in microtubes, then frozen at −20°C until analysis.

Serum activities of gammaglutamyltransferase (GGT; modified Szasz method) and alkaline phosphatase (ALP; modified Bowers and McComb method) and serum concentrations of total protein (TP; biuret method) were analyzed using a semiautomatic spectrophotometer (Labquest, Labtest Diagnóstica, Lagoa Santa, Minas Gerais, Brazil) with light of appropriate wavelength for each test using a set of commercial reagents (Labtest Diagnóstica, Lagoa Santa, Minas Gerais, Brazil).

Protein fractionation of colostrum and serum samples for the determination of immunoglobulins and lactoferrin was determined using sodium-dodecyl sulfate polyacrylamide gel electrophoresis (SDS-PAGE), according to the technique described by Laemmli (10). The concentrations of these proteins were determined using computerized densitometry (Shimadzu CS-9301 PC, Tokyo, Japan). As a reference, a marker solution with different molecular weights was used in addition to the purified bovine IgG protein (Sigma, St Louis, MO, USA).

### Statistical Analysis

To observe the effects of treatment (dam colostrum or calf blood) throughout the experimental period (0, 24, 48, and 72 h after calving) and the respective interactions (treatment × time), the parameters were analyzed as repeated measures using the MIXED procedure in SAS version 9.4 (SAS/STAT, SAS Institute Inc., Cary, NC, USA). In cases wherein the premise of specificity was not met (P < 0.05), the probabilities of time (*P*-value of time) and interactions of treatment and time (*P*-value for treatment × time) were corrected using the Greenhouse–Geisser epsilon formula. Comparison between treatments at each time point (*P*-value) was performed using the least-squares means (LS means) test following repeated measures analysis.

Correlation analysis among all response variables was performed using the CORR RANK procedure in SAS. Relationships among the studied parameters were determined from these correlations. To predict 24-h-old buffalo calf IgG or colostrum IgG (dependent variables), non-polynomial simple regression equations were formulated using other responses as independent variables (colostrum IgG, colostrum TP, and 24-h-old calf TP). For this, the GLM procedure of the SAS program was used using the LS means methodology.

The regression and correlation coefficients obtained were considered high only when determination coefficients (*R*^2^) ≥ 0.70 and correlation coefficients (r) ≥ 0.50. P was considered statistically significant at ≤ 0.05. Graphics were created using Sigmaplot version 12.0 (Systat Software GmbH, Erkrath, Germany).

## Results and Discussion

**Figure 1:**
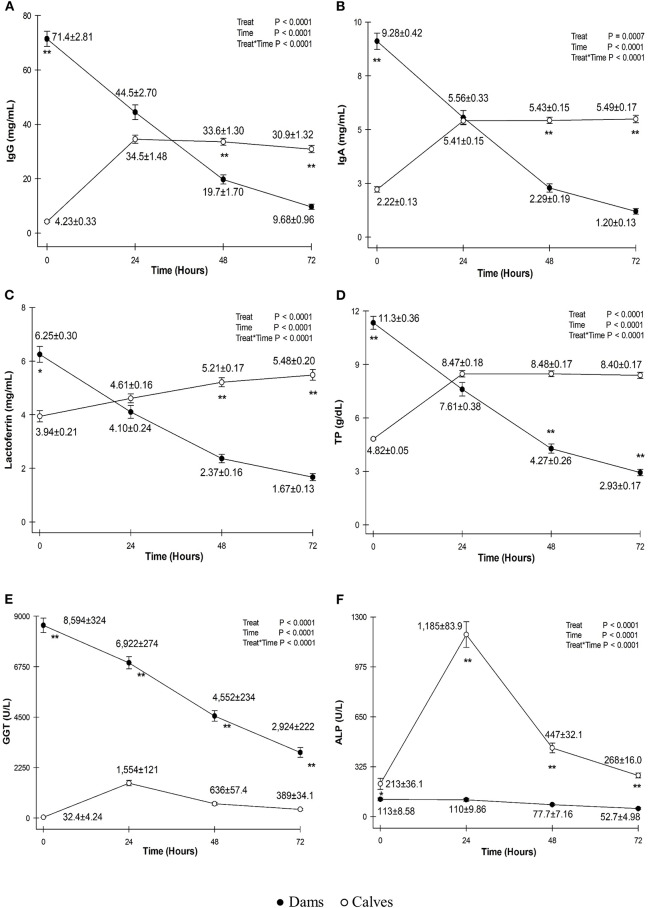
Mean ± standard deviation of serum concentrations of immunoglobulin G (IgG) **(A)**, IgA **(B)**, lactoferrin **(C)**, total protein (TP) **(D)** and serum activities of gammaglutamyltransferase (GGT) **(E)** and alkaline phosphatase (ALP) **(F)** in the colostrum of dams (black circles) and serum of calves (white circles) before colostrum intake and at 0, 24, 48, and 72 h. *P < 0.01; **P < 0.0001.

Serum concentrations of IgG, IgA, lactoferrin, and TP and serum activities of GGT and ALP in the colostrum and blood of calves are shown in Figure 1.

PIT is determined according to colostrum quality and calf absorption. The moment of colostrum intake, the method of administration, and the volume of colostrum are linked to calf absorption, whereas nutrition, breed, calving interval, dry-off period, and vaccination are determinants of colostrum quality. Therefore, IgG concentrations in both colostrum and calf sera should be estimated regularly to test compliance with colostrum management (6, 11).

The most common methods to assess the PIT status in domestic animals, measuring direct IgG concentrations, are single radial immunodiffusion (SRID), used as the gold standard, and enzyme-linked immunosorbent assay (ELISA), even if these methods are not feasibly correlated (12). SDS-PAGE, refractometry, and sodium sulfite or zinc sulfate turbidity test are used to estimate serum IgG concentration based on the overall protein concentration (13). Moreover, there are new on-farm methods, such as the split trehalase IgG assay (STIGA), that are still undergoing validation testing (14).

The mean serum concentration of IgG in the colostrum of dams was 71.4 ± 2.81 mg/ml on first milking, which was a high value considering that 50 mg/ml is used as the standard for good quality colostrum in bovine cattle, as no standard for buffaloes is available (11). This result was higher than those reported for Egyptian buffaloes using SRID (33.20 mg/ml) (15) and Murrah buffaloes using indirect ELISA (54, 51.7 ± 5.99, and 57.9 ± 5.71 mg/ml) (4–6). After calving, IgG concentration decreases as colostrum finishes turning into milk at approximately the fifth day after calving (15).

These results can be linked to the vaccination schedule of the farm, which must be responsible for the high IgG concentrations in the colostrum derived from the dam's bloodstream. Additionally, all buffaloes had good nutrition and had their dry-off period respected, which is important for females to regain body condition until the end of pregnancy (16) and to prepare for all the metabolic challenges inherent in the transition period (17, 18), and so that the mammary gland can recover from the previous lactation. This is essential in primiparous dams, which experience significant physiological changes during their first lactation. Moreover, the division of the females between categories enabled the proper feeding of heifers. Without the dominance of older dams, heifers could eat an amount adequate for growth, calf development, and boosting their immune system status, culminating in high serum concentrations of IgG in colostrum.

Calves were born hypogammaglobulinemic (4.23 ± 0.33 mg/mL) and after suckling colostrum; at 24 h, serum IgG concentrations increased to an average of 34.5 ± 1.48 mg/ml. Similar values have been reported at 24 h in Mediterranean buffalo calves (31.0 ± 2.4 mg/ml) (19) and descendants of Murrah heifers at 24 h (35.3 ± 8.58 mg/ml) (3). Moreover, the values are higher than those measured by indirect ELISA at 6–12 and 12–18 h after first feeding [11.7 ± 0.75 mg/ml (20) and 11.2 ± 0.7 mg/ml (21)].

The high serum concentrations of IgG in calf blood in this study can be attributed to the short time between calving and first feeding (<4 h after birth). As shown in Figure 1, there is a point of inflection at 24 h that is common for both colostrum and calf blood serum concentrations, with the IgG concentrations in calf blood surpassing the amount of IgG in colostrum after 48 h. Because calves are allowed to nurse the dam freely, the volume of colostrum ingested by the calf is not limited by human workforce or proper colostrum management, resulting in high serum concentrations of IgG that will endure until the first month of age (3).

The correlation between dam colostrum parameters at calving and calf blood parameters at 24 h is summarized in Table 1. A correlation index of 0.46357 (*P* < 0.0001) was found within dam colostrum IgG at parturition and serum concentrations of IgG at 24 h in calf sera.

**Table 1:** Correlation between colostrum parameters at calving and serum concentrations parameters of buffalo calves at 24 h.

****	**IgGDam**	**TPDam**	**GGTDam**	**ALPDam**	**IgGCalf**	**TPCalf**	**GGTCalf**	**ALPCalf**
IgGDam								
*r*	1.00000							
P								
TPDam								
*r*	0.97376	1.00000						
P	<0.0001							
GGTDam								
*r*	0.35266	0.37227	1.00000					
P	0.0046	0.0027						
ALPDam								
*r*	−0.01078	0.04029	0.22487	1.00000				
P	0.9310	0.7462	0.0652					
IgGCalf								
*r*	0.46357	0.38910	0.14147	0.02769	1.00000			
P	<0.0001	0.0012	0.2535	0.8174				
TPCalf								
*r*	0.39218	0.31104	0.07702	−0.02464	0.92647	1.00000		
P	0.0011	0.0110	0.5356	0.8372	<0.0001			
GGTCalf								
*r*	0.16585	0.14650	0.49548	0.06960	0.39202	0.34779	1.00000	
P	0.1867	0.2442	<0.0001	0.5670	0.0008	0.0032		
ALPCalf								
*r*	0.34998	0.29454	0.21756	−0.02902	0.26533	0.04735	0.17515	1.00000
P	0.0040	0.0164	0.0770	0.8102	0.0253	0.6950	0.1470	

It has been established that PIT evaluation is better performed between 24 and 72 h after first feeding. However, measurements within 1 week or even 10 days after birth can be performed with reliable confidence (11, 22). A cutoff point of 10 mg/ml for IgG is recommended to determine PIT in dairy bovine calves (23); however, a more realistic standard of 20–25 mg/ml has recently been established for dairy calves based on the improvements made in colostral feeding practices in the dairy industry (24). Unfortunately, there is no cutoff point for PIT in buffalo calves.

Nevertheless, a more efficient PIT means a better average daily gain (ADG) and heavier calves at 30 days in buffaloes (19), with the same positive correlation between serum IgG levels and ADG reported in pre-weaned Holstein calves (25). It is important to strive for higher PIT levels because it clearly enhances production efficiency on dairy farms, contrary to a previous report that there is no benefit in surpassing the recommended IgG threshold for avoiding FPIT (23).

The mean IgA concentration in dam colostrum soon after calving was 9.10 ± 3.89 mg/ml. The present study had higher values at 24 h than those reported in Murrah buffaloes using ELISA (3.22 mg/ml) (4). This difference is probably due to the data sample (*n* = 72 vs. *n* = 8 dams) and method of quantification (SDS-PAGE vs. ELISA).

Calves were born with low serum levels of IgA (2.23 ± 0.14 mg/ml). After feeding, at 24 h, newborns exhibited an average IgA level of 5.41 ± 0.15 mg/ml. IgA acts to prevent infections through agglutination of microorganisms, binding to the intestinal wall receptors and, together with IgG, is essential for providing neonates with immunological protection during at least the first 2–4 weeks of life (26, 27).

The mean lactoferrin concentration in dam colostrum was 6.25 ± 0.30 mg/ml on first milking, which is higher than that of Egyptian dams (1.08 mg/ml) soon after calving (15). Lactoferrin is present in buffalo milk in lower quantities (0.05–3.40 mg/ml) (28) and is an indicator of the health of mammary glands. It acts as an important immunomodulator because it binds to iron molecules, impeding their availability to bacteria because they bind to cell-surface receptors and facilitate iron absorption (27).

Serum concentrations of lactoferrin in calves exhibited an increase after first feeding, with an average of 4.61 ± 0.16 mg/ml, and continued to increase until the third day (5.48 ± 0.20 mg/ml), as it continued to be absorbed differently from what occurs with Ig's (Figure 1). Lactoferrin, when absorbed and in plasma, is called “transferrin” and is responsible for iron transportation. In addition, it has other functions including antiviral and antibacterial activities, and it also acts as a growth factor (27).

**Figure 2:**
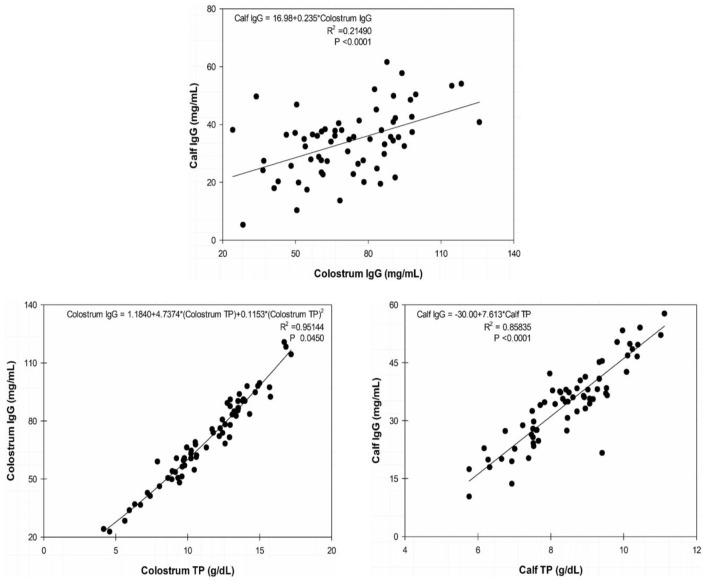
Scatter plots of calf immunoglobulin (Ig)G at 24 h × colostrum IgG at 0 h; colostrum IgG × colostrum total protein (TP) at 0 h; and calf IgG × calf TP at 24 h. The line represents the tendency of the data determined by simple linear regression on calf blood serum and quadratic regression on dam colostrum.

We found high correlation indexes between both colostrum IgG and TP at the first milking (0.97376 [*P* < 0.0001]) and between serum concentrations of IgG and TP in calf sera at 24 h after birth (0.92647 [*P* < 0.0001]). Determination indexes are presented in scatter plots in Figure 2.

TP is a reliable indirect parameter used to evaluate PIT because TP concentrations have high sensitivity and specificity for the detection of FPIT. Due to the small variation in serum albumin concentrations in newborns, the increase in TP concentrations is almost exclusively due to the absorption of Ig's present in colostrum (29).

Dam colostrum had an average TP concentration of 11.3 ± 2.4 g/dl soon after parturition. TP dynamics, presented in Figure 1, show a turning point both in colostrum and calf blood at 24 h, with the TP concentrations in calf sera surpassing those from colostrum and without a significant difference between them (*P* ≤ 0.05). Mean serum TP concentration in calves before colostrum feeding was 4.82 ± 0.05 g/dl and, at 24 h of age, reached an average concentration of 8.47 ± 0.18 g/dl, which was above the recommended threshold for an efficient PIT in dairy bovine calves at 24 h (5.8–6.3 g/dl) and consistent with serum TP concentrations reported in buffalo calves born from multiparous buffaloes (3, 24).

In water buffaloes, GGT determines the availability of amino acids for milk protein synthesis during lactation and is present in great quantities during colostrogenesis, accumulating until first milking. In this manner, it has positive correlation (86%) with IgG values measured electrophoretically and is recommended to evaluate colostrum quality from buffalo dams (30, 31). In addition, GGT levels demonstrated the best balance between sensitivity and specificity when compared with IgG measured by ELISA using SRID as the gold standard (29).

Blood serum activities of GGT in calves exhibited an increase between 0 and 24 h (32.4 ± 4.24 to 1,554 ± 121 U/L) following the ingestion of the enzyme present in high quantities in colostrum at the time of calving (8,594 ± 324 U/L). In the following days, serum activities in calf sera decreased, as GGT was degraded over time by the calf intestine (Figure 1).

A correlation of 0.49548 (*P* < 0.0001) was found between colostrum serum activities of GGT at first milking and blood serum activities in calves 24 h after birth, which confirms the origin of high serum activities of GGT in calves in the first week of life (3).

The serum activity of ALP in calf blood was low soon after birth and increased at 24 h, with an average of 1,185 ± 83.9 U/L. This could not be due to absorption of ALP from colostrum because it had low levels since the first sampling (113 ± 8.58 U/L).

There was no correlation between the serum activity of ALP on first milking and calf blood at 24 h. Moreover, a weak correlation was found between the ALP content and the serum concentrations of IgG at 24 h (0.26533 [*P* < 0.05]). The non-correlation of this enzyme with IgG concentrations has been previously reported (30).

The increase in serum activity of ALP might be due to the isoenzyme of bone origin because there is an increase in the activity of bone isoenzyme ALP in animals with high osteoblastic activity, as in neonates (32).

## Conclusions

PIT was successfully achieved in the studied calves. Colostrum from buffalo dams had high IgG concentrations before first milking, which resulted in high IgG concentrations in the calf blood 24 h after birth. Moreover, concentrations of TP served as reliable indirect parameters to evaluate colostrum quality and PIT in buffalo calves. ALP is not recommended for assessing PIT.

Calves had IgG concentrations well above the utilized cutoff point in dairy calves. We emphasize the importance of a vaccination schedule and proper nutrition for dams. Additionally, it is important for calves to nurse directly and freely from the dam. The presence of the dam with the newborn in the first days after calving is also important.

## Data Availability Statement

The datasets generated for this study are available on request to the corresponding author.

## Ethics Statement

This research project was evaluated by the Ethical Committee on the Use of Animals of FCAV/UNESP, Jaboticabal Campus (São Paulo, Brazil) and approved under protocol number 17.366/16.

## Author Contributions

DCS participated in the conception and design of the study, sample collection, laboratory analysis, data interpretation, and coauthored the manuscript. DGS participated in the conception, design and coordination of the study, laboratory analysis, and coauthored the manuscript. LCCF and LCF participated in laboratory analysis and coauthored the manuscript. OB participated in the conception and design of the study, contributed resources, and coauthored the manuscript. BM participated in the conception and design of the study, performed the statistical analysis, and coauthored the manuscript. RV and JF participated in the conception, design, coordination of the study, and coauthored the manuscript. All authors read and approved the final manuscript and study coordination.

## Conflict of Interest

The authors declare that the research was conducted in the absence of any commercial or financial relationships that could be construed as a potential conflict of interest.
